# Immunogenicity and tolerance induction in vascularized composite allotransplantation

**DOI:** 10.3389/frtra.2024.1350546

**Published:** 2024-02-13

**Authors:** Jiahui (Angela) Sun, Aisha Adil, Felor Biniazan, Siba Haykal

**Affiliations:** ^1^ Latner Thoracic Surgery Laboratories, University Health Network, Toronto General Hospital, University of Toronto, Toronto, ON, Canada; ^2^ Institute of Medical Science, Temerty Faculty of Medicine, University of Toronto, Toronto, ON, Canada; ^3^ Division of Plastic & Reconstructive Surgery, Department of Surgery, University of Toronto, Toronto, ON, Canada

**Keywords:** vascularized composite allograft (VCA), immunogenicity, graft rejection, tolerance induction, tissue engineering

## Abstract

Vascularized composite allotransplantation (VCA) is the transplantation of multiple tissues such as skin, muscle, bone, nerve, and vessels, as a functional unit (i.e., hand or face) to patients suffering from major tissue trauma and functional deficits. Though the surgical feasibility has been optimized, issues regarding graft rejection remains. VCA rejection involves a diverse population of cells but is primarily driven by both donor and recipient lymphocytes, antigen-presenting cells, macrophages, and other immune as well as donor-derived cells. In addition, it is commonly understood that different tissues within VCA, such as the skin, elicits a stronger rejection response. Currently, VCA recipients are required to follow potent and lifelong immunosuppressing regimens to maximize graft survival. This puts patients at risk for malignancies, opportunistic infections, and cancers, thereby posing a need for less perilous methods of inducing graft tolerance. This review will provide an overview of cell populations and mechanisms, specific tissue involved in VCA rejection, as well as an updated scope of current methods of tolerance induction.

## Introduction

Trauma, burns, tumor removals, and congenital malformations can result in large volumetric soft tissue loss and present several clinical challenges. Patients have compromised quality of life, functional deficits, and, in some cases, permanent disability (1, 2). Such conditions necessitate complex surgical reconstruction. Current autologous approaches entail using local tissue rearrangements, flap transfers, and grafts; however, these options are limited by donor tissue availability, donor site morbidity, and the need for multiple surgical interventions (3). Functional and esthetic restoration can also be limited with such approaches. Vascularized composite allotransplantation (VCA) is an emerging reconstructive option that entails transplanting multiple, heterogenous tissues (e.g., skin, nerve, muscle, bone, vessels) as a functional, composite unit from donor to recipient. VCA holds strong promise for patients with severe defects and advanced reconstructive needs not amenable with standard reconstruction. Along with esthetic restoration, VCA also offers potential for restoration of functional, sensory tissues necessary for adapting and reintegrating transplanted tissues for functional use in recipients. Examples of complex tissues include face, extremities (e.g., hand, leg), genitourinary tissues, and trachea among many others (4). Despite its potential, VCA has multiple challenges that prevent its widespread clinical use.

A longstanding challenge for functional restoration of VCA grafts is reinnervation. While nerve repair and coaptation is a major requirement of VCA to enable functionalization of tissues dependent on motor and sensory function, it has also been found to promote immuno-inflammation post-transplantation (4, 5). Hence, potential avenues for adjunct treatments to address this neuroinflammatory challenge in VCA are of interest. For example, the commonly employed immunosuppressant tacrolimus has demonstrated beneficial neuroprotective effects (5). Further, composite grafts are more complex given the multi-tissue composition with varying immunogenic profiles between the different tissue types. With the varying functions and forms of these tissue types, the alloimmune responses have different presentations and levels of rejection that can be difficult to address (2, 6, 7). To mitigate rejection and ensure survival and maximal tolerance of VCA grafts, potent and long-term immunosuppression regimens are needed. These regimens are modeled after those used in solid organ transplants (SOTs) and are not tailored for the complexity of VCA grafts (8). These drugs, however, can have severe and potentially life-threatening side effects for patients including increased risk of opportunistic infections, malignancies, organ toxicity and dysfunction, and reinnervation challenges that can impact grafts’ functionality (6, 8, 9). To address these shortcomings, devising methods for regulating immunogenic responses and inducing immune tolerance can significantly advance the clinical implications of VCA. With this context, tolerance induction and reducing the intensity of immunosuppressive protocols and mitigating graft rejection have become increasingly active areas of investigation.

Herein, the present review will focus on the current understanding of acute and chronic rejection mechanisms in VCA, the immunogenicity of composite grafts, and how different immune cell populations contribute to VCA rejection. Methods and current progress on tolerance induction for VCA are also reviewed. Experimental animal models ranging from both small and large animals are discussed throughout for how they are used to examine the different types of immunosuppressive protocols, their technical feasibility, and potential tolerance induction methods (10). The pertinent next steps in the field from both scientific and clinical perspectives and implications for clinical translatability are also discussed.

## VCA rejection

Similar to SOT, graft rejection in VCA is a significant concern. However, no non-invasive/systematic assays are available for the monitoring of cell markers that indicate VCA graft rejection (11). Fortunately, VCAs have the advantage of direct observation of the graft, allowing for easier identification of rejection. For clinical purposes, VCA rejection has been categorized into acute (occurring between days to months following the transplant) and chronic rejection (which can occur years after the transplant). Currently, acute rejection is more commonly reported, with an incidence rate of over 89%, compared to an incidence rate of 11% in chronic rejection of face and upper extremity transplant patients (12–14). It is unclear why acute rejection has a higher incidence rate in VCA than in SOT, but it has been hypothesized that it may be partly due to easier identification of rejection in VCA through visual monitoring (12). Though most episodes of acute rejection are reversible with prompt treatment, the number of acute rejection occurrences within a patient may predict or even contribute to the onset of chronic rejection and graft loss (15).

### Acute rejection

Acute rejection occurs days or months following the transplant procedure, when an intense and rapid but typically reversible immune response is triggered against the transplanted tissue. This process predominantly involves cell-mediated rejection, which occurs through cytotoxic cells such as T cells. Therefore, current clinical immunosuppression regimens such as tacrolimus, steroids, and mycophenolate mofetil (MMF) predominantly target T cells (16). In addition, donor-specific antibodies (DSA) produced by plasma cells can also direct immune cells to attack the graft, and this is known as antibody-mediated rejection (AMR) (3). A Banff classification of AMR in kidney transplantation was defined in 2011, but no diagnostic criteria exist for VCA (17). Studies have proposed non-invasive biomarkers of rejection such as matrix metalloproteinase 3 (MMP3), CCL18, and CD1. Proteins of the MMP family are involved in the breakdown of extracellular matrix (18). MMP3 has been found to increase in levels following transplantation and peak during severe rejection of face and upper extremity, skin-containing VCA recipients, suggesting association between metallopeptidase activity and severe acute rejection (19–21). In addition, skin biopsies from bilateral limb transplantation show that antigen-presenting cells (APCs) expressing the chemokine CCL18, which binds to the CCR8 receptor, can recruit more T cells to the skin graft, resulting in accelerated skin rejection (22). Despite the need for further evidence, these biomarkers hold potential as candidates for non-invasive diagnostics in acute rejection of VCA.

The current standard monitoring and diagnostic for acute VCA rejection includes a visual inspection as well as skin biopsies. Older practices involved using additional distant transplants of the donor skin for monitoring and biopsy purposes (23). It was later observed that such non-vascularized grafts lost their monitoring potential over time due to differences in cell trafficking and immune responses as a result of different vasculature. As such, vascularized sentinel flaps placed in discreet locations such as the chest and the groin have been utilized instead (24). Upon visual examination, acute rejection is characterized by redness, maculopapular rash, edema, induration, lesion, and/or oral ulceration (12, 25). Since the clinical appearance of early rejection is not entirely specific and highly subjective, a skin biopsy is obtained and evaluated histologically using the Banff classification of skin rejection established in 2007 (26). The Banff classification characterizes the degree of rejection in terms of localization and intensity of the inflammatory infiltrate and presence of target cell injury (25). Five grades of rejection severity are defined using features including inflammatory cell infiltration with the involvement of epidermis and/or adnexal structures, epithelial apoptosis, dyskeratosis, and necrosis (Table 1) (27).

**Table 1:** The Banff 2007 working classification of skin-containing VCA pathology (27).

Rejection grade	Histopathologic characteristics
Grade 0	No or rare cellular infiltration
Grade I	Mild perivascular dermal infiltration, no involvement of the epidermis
Grade II	Moderate to severe perivascular infiltration, potential mild epidermal, and/or adnexal involvement
Grade III	Infiltration into epidermis, epithelial apoptosis, dyskeratosis, and/or keratinolysis
Grade IV	Frank necrosis of epidermis or other skin structures

### Chronic rejection

Chronic rejection is the process of progressive loss of function in the graft, mediated by immune and potentially non-immune mechanisms (23, 27). It is typically irreversible and results in the gradual loss of function as well as accelerated aging of the graft (3).

The monitoring and diagnosis of chronic rejection is poorly defined due to the lack of understanding of chronic rejection as well as low incidences of chronic rejection at the moment. Neither the 2007 Banff classification of skin rejection nor the 2011 Banff classification of AMR in kidney transplantation include characteristics of chronic rejection in VCA (17, 27). There have been efforts to establish a definition of chronic rejection in VCA patients between the American Society of Reconstructive Transplantation and the International Society of Vascularized Composite Allotransplantation since 2018, but a specific diagnostic system has yet to be established (28). Despite the lack of standardized characteristics, there have been documented histologic and clinical features of chronic rejection including vascular narrowing, loss of adnexa, skin and muscle atrophy, fibrosis of deep tissue, myointimal proliferation of vessels, and nail changes (27). In general, graft vasculopathy seems to be a hallmark of chronic rejection in VCA (29). It is characterized by concentric vascular narrowing with intimal hyperplasia and adventitial scarring, ultimately resulting in necrosis of the graft (29, 30). Interestingly, studies have found isolated damage to skin or vascular structures (28). As such, it is hypothesized that there may be two phenotypes of chronic rejection, one that involves chronic immune-mediated arteriosclerotic change of the vasculature, and one that involves cellular rejection mechanisms and predominantly affects the skin (3).

Though limited, there have been studies in animal models such as rats and non-human primates, where chronic rejection was induced by periodically discontinuing or completely weaning animals from the immunosuppression treatment. An orthotopic hindlimb transplant model of VCA in rats was used in a study conducted by Unadkat et al. in 2010 to study the effects of multiple acute rejection episodes, each completely reversed with a combination treatment of cyclosporine (CsA) and dexamethasone. Results at postoperative day (POD) 90 showed that animals that went through repeated acute rejection demonstrated significant vascular lesions along with skin and muscle atrophy, upregulation of profibrotic gene expression, and fibrosis when compared to animals that did not go through acute rejection. Muscle atrophy with macrophage infiltration was seen after a few acute rejection episodes, with vasculopathy observed later. In addition, allograft bone was noted to be sclerotic and weak. This study demonstrated the first evidence of composite tissue vasculopathy and degeneration (CTVD) in VCA (15). A more recent study conducted by Puscz et al. in 2020 applied a rat allogeneic hindlimb transplant model used to study chronic rejection in solid organ transplants to imitate chronic rejection in VCA and identify potential markers (30). Rats were treated with immunosuppression only in case of acute skin rejection. This study was able to induce and detect significant intimal proliferation. In addition to allograft vasculopathy, the results also showed migration of immunological cells such as CD4+ and CD68+ cells, which are known to be major participants in the tissue remodeling processes during chronic rejection. This study also identified CXC ligands 9–11 as potential markers of chronic rejection through microarray analysis and subsequent qPCR (28). Elevated levels of CXCL11 was found in chronically rejected human face allografts (31), urinary CXCL9 and 10 were identified as potential biomarkers for subclinical rejection in kidney transplants (32), and CXCL9 was associated with rejection of liver allografts as well (33). Therefore, CXCL9, 10, and 11 may serve as potential biomarkers for chronic rejection in VCA. Despite many proposed hypotheses, there are still many uncertainties regarding the cellular mechanisms of chronic rejection in VCA, which require further evidence.

Acute and chronic rejections in VCA present formidable challenges in maintaining graft survival. The occurrence of acute and chronic rejection in VCA can vary based on several factors, though acute rejection is generally more common and tends to occur earlier in the post-transplant period compared to chronic rejection. Currently, acute rejection demands immediate attention and robust immunosuppressive interventions to prevent irreversible damage. Chronic rejection, with its insidious nature, necessitates a long-term perspective, focusing on sustained graft function and minimizing the impact of vasculopathy and fibrosis. As the work in exploring the immunogenicity of VCA progresses, understanding the cellular mechanisms in graft rejection can give valuable insight in how to effectively mitigate rejection.

## Cellular mechanism of acute rejection

The strongest immune system activation occurs early following transplantation. The transplant procedure will result in a combination of ischemic injury that triggers innate immunity as well as the presentation of donor antigens by APCs to recipient T cells in the lymphoid organs, activating adaptive immunity (12). Though predominantly orchestrated by T-cell subpopulations, other cell types such as endothelial cells (ECs), B cells, and natural killer (NK) cells, have been shown to be involved in the process of acute graft rejection as well (3).

### T cells

T cells are a major component of adaptive cell-mediated immunity and the main cell population involved in VCA rejection. They express highly organized molecular complexes divided into the central, peripheral, and distal regions on the cell surface to aid their functions. The central region contains the T-cell receptor (TCR) complex, co-stimulatory, and co-inhibitory molecules, which are the primary and secondary activation signals. The peripheral region is composed of adhesion molecules LFA-1-ICAM-1 and CD2-LFA-3, which support the binding between cells. The distal zone contains F-actin and phosphatase CD45 (34). There are two main subpopulations of T cells based on the glycoprotein expressed on their respective TCR: CD8+ cytotoxic T lymphocytes (CTL) and CD4+ T-helper (T_h_) cells.

### T-cell activation

**Figure 1:**
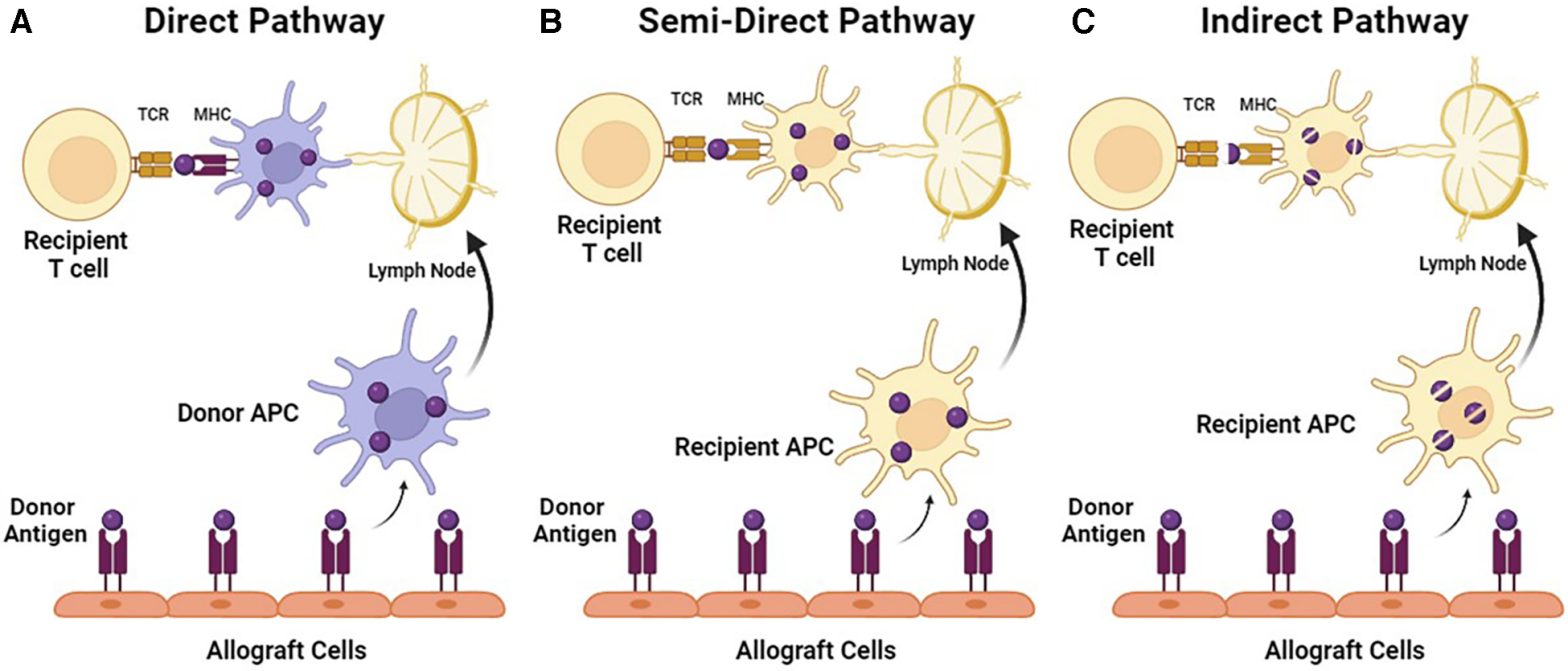
Pathways of T-cell allorecognition. (**A**) In direct pathway, MHC class I and II alloantigen is recognized as intact proteins on the surface of donor APC by CD4+ or CD8+ recipient T cells. (**B**) In a semi-direct pathway, MHC alloantigen is acquired by recipient APC, but presented as an intact protein. (**C**) In indirect pathway, MHC alloantigen is acquired by recipient APC and presented as peptide fragments.

Both CD4+ and CD8+ T cells were noted to be present in rejected skin grafts as early as 1982. Overall, CD8+ T cells were more abundant in the epidermis and hair follicles, while CD4+ T cells were predominantly found in the graft dermis and graft bed (35). It was proposed that rejection can occur through a direct pathway of epithelial injury mediated by CD8+ CTLs, or an indirect pathway of T-cell-mediated endothelial microvascular injury (35). Antigen recognition can occur directly and indirectly as well. In the direct pathway of antigen recognition, CD4+ and CD8+ T cells recognize intact major histocompatibility complex (MHC) class II and class I alloantigen, respectively on the donor APCs (36). This concept has led to the passenger leukocyte theory, which proposes that allograft rejection is triggered by direct pathway recognition of donor dendritic cells (DC) that migrated from the allograft to host secondary lymphoid tissue (36). The indirect pathway is the conventional mechanism of presenting bacterial/virus antigens, where dendritic cells acquire a foreign antigen through endocytosis and process it into peptide fragments. The fragments are then presented on their cell surface as MHC molecules (36). The T cells will bind via the TCR and recognize the antigen as non-self, resulting in an MHC mismatch, and subsequently, a signaling cascade to activate three essential transcription factors: NFAT, AP-1, and NF-κB (34). In addition, it has been demonstrated that intact antigen could also be transferred between different cell types, suggesting the possibility of a semi-direct pathway where the direct pathway of T-cell recognition may occur in recipient dendritic cells (37) (Figure 1).

T cells express co-signaling receptors that, upon binding, are not able to activate T cells on their own but can significantly amplify or reduce the signaling induced by the TCR complex. They are strictly regulated due to their role in directing T-cell activation, expansion, and differentiation and ultimately T-cell fate. A co-stimulatory molecule is also required for T-cell activation. Upon activation, T cells begin proliferation, differentiation, as well as expansion of the differentiated T-cell population. Co-stimulatory molecules display great diversity in expression, structure, and function, with the most well described being CD28. It is constitutively expressed in both CD4+ and CD8+ T cells and binds to ligand B7-1 and B7-2, which is expressed on activated APCs (38). A number of co-stimulatory receptors including ICOS, CD226, OX-40, 401BB, and GITR have been identified to date (39). In contrast, co-inhibitory molecules control and contract the expanded T-cell population, thereby maintaining balance between tolerance and immunity. There are multiple co-inhibitory receptors including CTLA-4, programmed death-1 (PD-1), TIM-3, TIGIT, and LAG-3. These receptors play a critical role in the regulation of T-cell function. Regulatory T (T_reg_) cells typically express CTLA-1 and PD-1, which promote the suppressive functions of T_reg_ cells (39). CTLA-4, or cytotoxic T lymphocyte-associated antigen-4, is structurally similar to CD28 and bind to the same ligands, B7-1 and B7-2, but with much higher affinity. This allows CTLA-4 to deplete CD28 ligands and subsequently prevent T-cell activation (39). The second co-inhibitory receptor PD-1 mediates inhibitory signals via ligands PD-L1 and PD-L2. It has been demonstrated that the PD-1 pathway plays an important role in autoimmune diseases (39).

### CD8+ T cells

CTL bind to target cells and induce apoptosis in target cells through the secretion of perforins and granzymes. Upon activation via MHC mismatch and co-stimulation, CTLs will differentiate to recognize donor-specific antigens and express tissue homing receptors that direct them to attack allograft tissue. It has been commonly believed that CTLs are reliant on T_h_ cells for their activation. However, it has been discovered that CTLs can differentiate into Tc1 and Tc2 cells and perform their cytotoxic functions possibly independent of T_h_ cells as well (40). In addition to their cytotoxic functions, activated CTLs also have the ability to produce high levels of pro-inflammatory cytokines including IFN-γ. This cytokine has been shown to upregulate the expression of MHC molecules and enhance alloantigen presentation on target tissues. In addition, IFN-γ also enhances inflammation to stimulate non-specific effector cells such as macrophages and NK cells (40). CTLs can also secrete chemokines to recruit leukocytes into sites of inflammation as well as amplify tissue inflammation by stimulating neutrophil degranulation and monocyte superoxide production (40). Moreover, donor T resident memory cells (T_RM_), identified by the markers CD69, CD103, and cutaneous lymphocyte antigen (CLA), can also be found within the allograft. This population can endanger graft survival and has been associated with early onset of VCA rejection on POD 5 (41).

### CD4+ T cells

T_h_ cells’ main function is to produce cytokines to activate or modulate CTL, macrophage, and B-cell functions (3, 34). Within the T_h_ cell subpopulations, T_h_ 1, T_h_ 17, and the regulatory T cells (T_reg_) cell populations should be noted for their effects in graft rejection/tolerance. T_h_1 secretes chemokines such as CXCL9, 10, and 11 are associated with chronic rejection, whereas T_h_ 17 generates a chronically inflammatory environment which can also contribute to allograft rejection (3, 42). T_reg_ cells (CD4+ CD25+ FoxP3+/CD127−) however, play a crucial role in establishing and maintaining immunological homeostasis and are essential for inducing tolerance to allografts (34, 43). They exert their immunosuppressive role through direct cell–cell interactions with target immune cells, or release immunosuppressive cytokines and anti-inflammatory molecules. Specifically, they can suppress the activation, proliferation, and effector function of reactive CD4+ or CD8+ T cells, NK cells, NK T cells, B cells, and APC (34). T_reg_ has demonstrated abilities to induce tolerance, one example being preventing skin allograft rejection in humanized mouse models (44). Currently, the utilization of T_reg_ in inducing tolerance in VCA is being heavily investigated.

Cellular infiltrates of human skin rejection samples in hand transplantation consist mostly of CD4+ and CD8+ T cells, with some B cells, macrophages, and histiocytes. Though CD4 expression did not correlate with cellular skin rejection, it did correlate with time after transplant in grade I rejection (45). It was found that stem cell-rich epidermal rete ridges, follicles, and dermal microvessels were the primary targets during acute rejection (46). Interestingly, over 90% of infiltrating lymphocytes in the epidermis and hair follicles were CD8+ and of donor origin, and lymphocytes of both donor and recipient accumulate near vasculature (12). In higher grade rejection, T cells are mostly activated with upregulation of genes associated with T-cell infiltration such as CD3D, CD3, CD4, CD8a; as well as T-cell co-stimulation such as CD28, TNFRSF4, ICOS, and TNFRSF9; T_h_1 chemokines such as CXCL9,10,11; and effector molecules including granzyme A, granzyme B, and granzyme K that are responsible for cytotoxicity (47). In terms of the antigen presentation pathway of T-cell activation, CD8+ T cells are activated predominantly through the direct pathway. While indirect pathway CD8 T-cell recognition of processed alloantigen is possible, it seems relevant only in the rejection of skin due to the processing and presentation of donor antigen by recipient endothelium (48). It has been demonstrated that the direct antigen-presenting pathway of CD4+ T cells occurs in the early stages of acute rejection and can contribute to allograft rejection by assisting the direct pathway of CD8+ T cells (48, 49).

### Epithelial and follicular stem cells

Stem cells are the unspecialized cells within the human body and bear the potential to differentiate into various cell types. Both epithelial and follicular stem cells have been proposed as the primary target of graft rejection (46). They are both characterized by high developmental capacity, making them multipotent stem cells. Specifically, epithelial stem cells can differentiate into keratinocytes. Follicular stem cells have shown greater development capacity and are able to differentiate into keratinocytes, melanocytes, mesenchymal cells, neurons, glial cells, and have shown to contribute to angiogenesis (50, 51). Cutaneous stem cells such as epithelial and follicular stem cells play an important role in skin regeneration and have a complex interaction with skin-resident and infiltrating immune cells such as T_regs_ and macrophages. T_regs_ surrounding the hair follicles have shown to affect hair growth through regulating follicular stem cell activity, whereas macrophages have the effect of suppressing follicular stem cell activity (52, 53). It has been shown that follicular stem cells upregulate the expression of MHC class I and II under pathological conditions, thus increasing the potential for interactions between follicular stem cells and immune cells. This mechanism may play a role in alloimmunity and be the cause of skin being the most targeted tissue in VCA rejection (3, 54). Biopsies from facial transplant recipients showed lymphocyte accumulation in in epithelial stem cell-rich regions, where intra-epidermal and intra-follicular cells were the main targets of donor-derived T cells (46).

### Endothelial cells

ECs form the single layer lining of all blood vessels and regulate exchanges between the bloodstream and the surrounding tissues (55). They also represent the interface between the allograft and recipient immunity following VCA, while acting as semi-professional antigen-presenting cells, making them one of the first targets of immune cells (56). They undergo direct and specific attack by MHC I and II recognizing NK cells, macrophages, and T cells, leading to endothelial cell lysis (56). Interestingly, ECs can activate and recruit lymphocytes through the upregulation of MHC class II and adhesion molecules such as E-selectin on the cell surface (46). Binding to the MHC II molecules in EC subsequently increases the secretion of IL-6, which then contributes to the expansion of T_h_17, which promotes inflammation, while reducing the immunosuppressive T_regs_ (57). E-selectin is responsible for trafficking leukocyte and allowing transendothelial migration of lymphocytes to the skin through the binding of CLA (3). In addition, increased IL-6 levels can initiate a signaling cascade that ultimately results in the disruption of the endothelial barrier crucial for the fluid, gas, and metabolic homeostasis of the graft (3). EC can further be involved in the cellular mediated rejection process in acute VCA rejection. This occurs as the allograft ECs present donor MHC molecules that are recognized by T cells, thereby triggering inflammatory pathways. Moreover, donor T_RM_, identified by the markers CD69, CD103, and CLA, can also be found within the allograft. This population can endanger graft survival and has been associated with early onset of VCA rejection on POD 5 (41). ECs have also demonstrated to be involved in AMR, though much less frequently in VCA than in SOT. C4d is a degradation product of the classic complement pathway and an immunological footprint of complement activation and antibody activity (58). The presence of C4d deposits is commonly associated with the presence of DSA in SOT and is a diagnostic criteria for classic AMR. Though it should be noted that unlike SOT, C4d deposits in VCA recipients were not associated with DSA, except for one confirmed case of AMR in which the C4d deposit was specific to the allograft and occurred in the presence of DSA (59, 60).

### NK cells, B cells, and macrophages

Other effector cells including NK cells, B cells, and macrophages can also participate in acute VCA rejection. NK cells are effector lymphocytes of the innate immune system that typically mediate anti-tumor and anti-viral responses (61). They express low affinity Fc receptors such as CD16 and can detect antibody-coated target cells, thereby inducing antibody-dependent cytotoxicity through the secretion of perforin and granzyme B (62). NK cells also interact with other cells such as mediating DC homeostasis via IFN-γ and TNF-α, prime T_h_1 cells through IFN-γ secretion, as well as increasing IgG and IgM antibody production and facilitating immunoglobulin class switching in B cells (61, 63). It has been identified that ECs can express chemokine CX3CL-1 and direct cells that express C-X3-C motif receptor-1 (CX3CR-1) into sites of inflammation. NK cells then secrete IFN-γ, future driving the expression of CX3CL-1 in EC, creating a feedback loop. In addition, IFN-γ induces T_h_1 responses and increases reactive oxygen species (ROS) levels, resulting in endothelial damage in allografts (64, 65).

B cells are myeloid cells originating from the bone marrow. They have a variety of functions such as differentiating into antibody-producing plasma cells, sustaining long-term humoral immune memory, serving as APCs, organizing formation of tertiary-lymphoid organs (TLO), and secreting pro- and anti-inflammatory cytokines (66). The DSA produced by B cells can be classified into preexisting or *de novo* DSAs, which occur within 3 months following transplantation. The presence of preexisting DSAs may predispose the patient to AMR. However, AMR is observed less frequently, and inconsistently, within VCA patients (3). In addition, there has not been concrete evidence for *de novo* DSAs and their effect on VCA rejection. However, it was noted that one face transplant recipient with AMR showed persistent presence of T_h_17, which has been associated in the induction of B-cell activation and differentiation (67). Furthermore, another face transplant patient who experienced chronic rejection showed dermal infiltration of CD20+ B cells, which formed TLO, which can further drive the proliferation and differentiation of autoreactive B cells (68, 69).

VCA infiltrating macrophages have been shown to produce IL-18 and ROS. IL-18 triggers the secretion of IFN-γ, whereas ROS can trigger pro-apoptotic B-cell lymphoma-2 (Bcl-2) family proteins and impair mitochondria functions (64, 70, 71).

## Tissue compartments of VCA

A VCA graft is often comprised of various types of tissues such as the skin, muscle, bone, vessels, nerves, tendons, and lymphatics. The concept of split rejection states that each has their own unique immune profile, causing them to interact with the recipient's immune system differently and reject at different times and intensities. These unique interactions are critical determinants of graft survival. Therefore, it is important to investigate the immunogenicity of each tissue type to understand the mechanisms of graft rejection and effectively find a method to induce tolerance.

### Skin

The skin is a major component of VCA and acts as the first layer of defense that our bodies have against the outside environment. Conspicuously, it has a complex population of immune cells and antigens that trigger immune responses. This sets the stage for immune interactions between the donor tissue and the recipient's immune system, resulting in potential graft rejection. The skin contains several important immunocompetent cells within the two major structures—the epidermis and dermis. The epidermis houses skin-resident immune cells such as Langerhans cells (LCs), which are a subset tissue-resident macrophages that acquire a phenotype and functions similar to DC upon further differentiation in the skin (72, 73). γδ T cells, which are innate immune cells that permanently reside in mice, as well as CD8+ T_RM_ cells can also be found in the skin (74). T_RM_ cells have been identified in several non-lymphoid tissues and their longevity can vary between different tissues. Specifically in the skin, they can appear after the resolution of inflammation and persist for over a year in mice (75, 76). The deeper layer of the skin, the dermis, contains other specialized immune cell populations such as DC subpopulations, macrophages, mast cells, and γδ T cells and innate lymphoid cells (ILCs) (77).

It has been assumed that the immunologic basis of skin rejection involved CD4+ and CD8+ T-cell populations of recipient origin (35). However, a recent study conducted by Lian et al. found that during active rejection, immune cells found near target cell injury presented an immunophenotype typical of resident memory T cells and were of donor origin (46). As such, CD8+ T cells of both donor and recipient origin are the primary effector cells targeting epithelial and follicular stem cells and microvascular endothelium (3). T cells found in the skin express the skin-homing receptors CCR4 and CLA, with the majority of the population composed of T effector memory (T_EM_) cells identified to be CD62l− and CCR7−, and circulate between the blood and skin, but not lymph nodes (23). In addition, a central memory T (T_CM_) cell population that are CLA+, CD62l+, and CCR7+ were found, suggesting their ability to circulate between the skin, blood, as well as the lymph nodes (23). Due to the abundance of memory T cells accumulated throughout the recipient's life within the skin, this poses the potential for a potent response from T_EMs_ following antigen presentation in VCA (23). The trafficking of memory T cells between the skin and draining lymph nodes have also been proposed as an explanation for the formation of TLO during rejection in VCA (11). TLOs are *de novo* lymphoid tissues containing clusters of T and B lymphocytes, and have been observed in chronic rejection of SOTs as well (11). Langerhans cells seem to maintain the immune homeostasis within the epidermis. They are not only efficient APCs, but have also been noted to induce the differentiation of naïve CD4+ T cells into T_h_2, T_h_17, and T_h_22, as well as priming/cross priming naïve CD8+ T cells (78–80). However, exposure to corticosteroids has been shown to increase the expression of TGF-β in Langerhans cells, thereby inducing the expansion of T_REGs_ in the skin (81). T_REGs_ typically exist within 5%–10% within the skin cell population and have an immunosuppressive effect in the skin as well. Skin-homing T_REGs_ express CLA, CCR4, and CCR6 in addition to typical T_REGs_ cell markers. They have been observed to proliferate in response to inflammation as well as downregulating inflammatory responses locally and en route to dermatotropic lymph nodes (e.g., inhibiting a stable binding between CD4+ T cells and APCs) (82, 83).

Historically, the skin has been viewed as the most immunogenic tissue within VCA due to the large donor-derived immune cell population carried within this tissue. As such, many studies solely focus on depicting the rejection mechanism of skin alone or in limb allografts (23, 47, 84, 85). However, recent studies in facial VCAs have identified the mucosal tissue as a main target of rejection as well, by consistently demonstrating more distinct microscopic changes indicative of acute rejection events when compared to skin biopsies, sometimes with a higher level of rejection in the mucosal tissues than in skin (3, 12, 86–89). As such, it has been proposed that the belief of skin being the primary target of acute rejection in VCA should perhaps be reevaluated, especially in the emergence of mucosa-containing allografts such as face, uterus and trachea.

### Vessels

Vessels are an essential component of VCA, allowing the flow of blood and nutrients to sustain graft survival. Unfortunately, VCA recipients experience aggressive allograft vasculopathy that result in graft failure (90). This is likely a result of initiating injury upon recovery or reperfusion, as well as immune-mediated EC and vascular wall injury. The innate immune system begins with a response involving neutrophils and macrophages, followed by a T-cell-driven alloimmunity, leading to the remodeling of vessels. In hand transplantation, inward remodeling of the vessels is observed with medial scarring and reduced matrix turnover. Inflammation, adventitial scarring, and intimal hyperplasia ultimately results in luminal stenosis, thereby obstructing blood flow (91). Furthermore, there may be an issue with lymphatic drainage in VCAs with significant areas of skin as a result of edema. This could lead to the insufficient trafficking of T and B lymphocytes, causing inflammation, adipogenesis, and fibrosis, ultimately contributing to graft rejection (92).

### Bone

A characteristic unique to VCA is the presence of vascularized bone within the graft. Interestingly, vascularized bone seems to contribute to a more tolerogenic environment for the graft than without bone, or even bone marrow cell infusions (93). However studies have shown that the tolerogenic effects of vascularized bone can be achieved using high graft to vascularized bone marrow mass ratios (94). This can result in a state known as “mixed chimerism,” in which the lymphohematopoietic system of the recipient comprises a mixture of both donor and recipient cells (95). Though it is important to note that such results have only been seen in animal studies, long-term stable mixed chimerism has not been achieved in human VCA recipients (11).

### Muscle and nerve

VCA, particularly in face and limb transplants, typically contains a significant amount of muscle tissue within the transplant. However unlike in SOT, there have been fewer signs of rejection observed in the muscle tissue in VCA. There have been studies exploring the rejection of muscle in VCA in both humans and porcine models (90, 96). Lymphocyte infiltration was noted between muscle fibers, as well as varying degrees of hypotrophy and fatty degeneration. However, changes are likely due to denervation rather than rejection (11).

## Tolerance induction in VCA

The ultimate ideal is creating a tolerant state within VCA recipients where recipients’ immune systems can accept both host and donor antigens as “self” (97). Tolerance refers to achieving a state of absent destructive immunologic responses in transplanted tissues. This definition can encompass additional components such as the lack of needing immunosuppression reagents, absence of lymphocyte infiltration, achieving donor-specific unresponsiveness, and lack of donor-specific alloantibodies (98). If donor-specific tolerance and functional recovery is achieved after immunosuppression discontinuation, VCA can be clinically used more broadly without the burden of immunosuppression and its effects (99, 100). Rodent models have been the primary models for percolating the basic mechanisms of alloimmune recognition and determining methods of tolerance induction; however, several studies have extended into large animal and non-human primate models as well. It must be noted that successful adaptation of tolerance protocols from murine models to larger animal models has been challenging, given differences in T-cell responses, MHC expression, and differences in mAb and co-stimulation blockade delivery across the models (97). Nonetheless, considerable progress has been made. Tolerance induction protocols may be classified across two categories: cell-based and pharmaceutical-based strategies. Cell-based approaches include using stem cells (e.g., bone marrow-derived cells) or immune cells (regulatory T cells, dendritic cells) along with a drug therapy regimen. Pharmaceutical-based therapies are currently the gold standard in VCA and are based off solid organ regimens. This entails an induction therapy followed by a multi-drug maintenance immunosuppression regimen (101). Many of these approaches have been promising in solid organ transplantation; however, in the applicability for VCA, these approaches can present long-term complications, especially when applying such tolerance induction approaches with deceased donor tissues. In the quest for achieving the ideal tolerant state, many of the strategies described have made considerable progress in achieving immunomodulation over long-term periods. To understand this further, different methodologies being explored for tolerance induction are discussed below.

### Mixed chimerism

The concept of mixed chimerism relies on using non-myeloablative conditioning and hematopoietic stem cell (HSC) transplantation, which can allow both donor and recipient immune cells to co-exist without adverse immunologic reactions (102). Mixed chimerism offers the advantage of mitigating graft-vs.-host disease and mitigating the lack of immunocompetence as otherwise observed with full chimerism (103). Chimerism induction is achieved using conditioning. Recipients are conditioned (e.g., using irradiation, cell-depleting agents) to control alloreactivity and vacate areas for donor bone marrow cells to engraft in. While both myeloablative and non-myeloablative conditioning exist, myeloablative conditioning has several disadvantages such as toxicity, being more aggressive, preventing autologous hematologic recovery, and is associated with more graft-vs.-host disease occurrences (97).

The mixed chimerism approach was first introduced in 1988 by Sykes and Sachs as an approach for transplantation tolerance (104). This approach was previously applied successfully in renal transplant tolerance studies in both animal models and clinical trials, with variability in the durability ranging between transient and stable mixed chimerism (13, 105–109). Similar protocols for VCA grafts rendered split tolerance with skin rejection (100). The first report of a full accepted VCA graft was that of a porcine fasciocutaneous MHC-mismatched graft where all components including the skin were accepted, providing evidence of tolerance induction across MHC mismatches in a large, pre-clinical VCA animal model. This entailed T-cell depletion using CD3 immunotoxin, 100 cGy total body irradiation, HSC transplantation, and cyclosporine A treatment for 45 days. VCA was performed 85–150 days post-HSC transplantation or simultaneously when inducing mixed chimerism within 56 h of HSC transplantation. In both conditions, following immunosuppression withdrawal, no rejection across any tissue compartments (including skin) was noted 115–504 days post-transplantation (102, 110). It must be noted that this tolerance using mixed chimerism was obtained across a haploidentical MHC barrier where MHC Class I and Class II antigens were shared between donor and recipient. This presents a unique challenge for applying such protocols for VCA—the VCA allografts obtained from deceased donors have full MHC mismatching in contrast to the successful tolerance seen in renal transplants that have single-haplotype MHC mismatches. Although Leonard et al. (110) provide a VCA-relevant proof-of-concept large animal model, it is only across single-haplotype MHC mismatch inapplicable for VCA on a clinical scale. Thus, understanding the role of MHC antigen matching for inducing tolerance using mixed chimerism is a critical step for moving forward with establishing tolerance in VCAs. Further studies applied the same protocol in MHC Class I-matched/Class II-mismatched and MHC Class I-mismatched/Class II-matched in porcine vascularized skin flaps. This helped clarify whether sharing MHC Class I or Class II antigens influences tolerance using mixed chimerism, where only MHC Class II-mismatched chimeras were found to be tolerant whereas MHC Class I-mismatched animals showed skin rejection (84). Lellouch et al. build on these findings and report stable mixed chimerism across Class I mismatch using a porcine osteomyocutaneous VCA model. To address the MHC Class I mismatch rejection, the authors adjusted their regimen by increasing irradiation, adding co-stimulation blockade (CTLA4-Ig/belatacept), anti-IL6R mAb (tocilizumab), and vascularized donor bone marrow as the source of cells. Stable mixed chimerism and tolerance were found in three of five recipients up to an endpoint of 400 days using this adjusted regimen across Class I-mismatched VCAs. To determine the immune competency, three split-thickness grafts were added to the recipients at 150 days post-transplantation from autologous, VCA donor, and third-party (MHC-mismatched to both donor and recipient) animal sources. Interestingly, only the autologous and VCA donor grafts were accepted, indicating donor-specific tolerance (111). Achieving mixed chimerism in a clinical setting for VCA using such regimens has yet to be attempted.

### Delayed tolerance

Delayed tolerance has been gaining traction for its potential application in VCA. Previously introduced for renal transplants, delayed tolerance involves delivery of conventional immunosuppression when recipients undergo transplantation followed by conditioning and delivering donor bone marrow transplants at a later time (112). This is a rather unique approach to achieving mixed chimerism, as inducing HSC engraftment and establishing mixed chimerism is attempted during the post-transplantation period whereby recipients’ immunologic milieu is largely pro-inflammatory and active toward donor antigens (112, 113). The concept of delayed tolerance however holds strong significance. Many of the conditioning regimens rely on conditioning days before transplantation, which makes these regimens selective for living donors only. To enable use of such tolerogenic protocols for deceased donors in the case of VCA, developing delayed tolerance induction protocols shows promise. This was investigated at Massachusetts General Hospital in non-human primates for solid organs, namely, kidneys and lungs, where a 4-month delay showed evidence of donor bone marrow transplantation engraftment, reduction in the inflammation, and evidence of mixed chimerism (97, 102). This approach was adopted for small and large VCA animal models. In 2020, Lellouch et al. attempted a delayed bone marrow transplantation in non-human primates (cynomolgus macaques) using hand or face VCA grafts. Interestingly, a 4-month maintenance immunosuppression rendered complications such as lymphoproliferative disorder in half of the recipients. A 2-month maintenance period was then applied which allowed recipients to progress toward the delayed tolerance induction timeline. However, authors report acute rejection in the 2–4 weeks post-transplantation and inability to induce mixed chimerism (113). Despite its many advantages, further work on establishing delayed tolerance for VCA remains elusive.

### T-cell depletion

Alloreactive T-cell depletion has shown potential in promoting tolerance induction. Prior to conducting VCA, T-cell depletion is conducted. After allotransplantation, the T cells are repopulated. This approach has been effective in promoting allograft acceptance in both animal models and in humans (114). In some cases, selective T-cell inhibition with cyclosporine administration has resulted in long-term allograft survival and evidence of mixed chimerism in rats (7, 115) In 2020, using a fully MHC-mismatched orthotopic mouse hindlimb VCA model, Oh et al. showed promising outcomes by demonstrating a long survival outcome of over 210 days with alloreactive T-cell depletion, co-stimulatory blockade (CoB), and total body irradiation treatment prior to surgery (116). In a large animal model, Leonard et al. used T-cell depletion in combination with total body irradiation and HSC transplantation whereby no rejection was observed in the period of 115–504 days post-transplantation (110). Much of T-cell depletion relies on combinatory use with other tolerance induction methods.

### Regulatory T-cell-mediated tolerance

Immune response regulation can also be modulated using regulatory T cells (Tregs), a T-helper cell subpopulation. While initially recognized as CD4^+^CD25^+^FoxP3^+^ T cells, the most common phenotype is CD4^+^CD25^+^CD127^−^FoxP3^+^. Tregs have become a mainstay in transplantation tolerance studies, given their ability to modulate self-tolerance and transplant rejection, though as such their use can have transient effects (101, 117). Tregs’ immunosuppressive mechanism of actions are multifold, where Tregs act through co-stimulatory pathways (e.g., CTLA4), anti-inflammatory agents (e.g., TGFβ), or cell–cell interactions with target immune cells (e.g., IL-2 removal) (101).

Many experimental studies have shown evidence of the association between Treg levels and improved VCA allograft survival. Bozulic et al. report that Tregs are important regulators in maintaining and promoting long-term composite tissue graft acceptance using rat hindlimb transplants where donor bone marrow transplantation, tacrolimus administration, and irradiation was used to induce tolerance. The skin of tolerant hindlimbs showed higher CD4^+^FoxP3^+^ infiltrates (118). In another study, Treg combined with vascularized bone marrow transplantation and co-stimulatory blockade and rapamycin treatment resulted in mixed chimerism and donor-specific tolerance in rats. Interestingly, the authors indicate that Treg infiltrates of recipient origin were detected, suggesting that Tregs potentially have a protective role for VCA rejection (119). Thus far, Treg regulation has not been extensively explored in tolerance induction studies using large MHC-mismatched VCA models. In 2022, Lellouch et al. reported no evidence of FOXP3^+^ cells in MHC Class I-mismatched swine VCA model (111). Previously, evidence of FOXP3^+^ cells was found in both porcine and canine haploidentical MHC-mismatched VCA models (110, 120). Using skin biopsies from human hand allografts six years post-transplantation, Eljaafari et al. report increased FOXP3^+^, TGFβ, and IL-10 expression indicating Treg presence in donor skin of allotransplant recipients (121). In an additional human hand VCA study, 104 skin biopsies from three recipients were taken over the course of 6 years post-transplant. FOXP3^+^ expression was found in tissues with severe rejection (122). Despite this progress, the mechanisms underpinning Treg immunomodulation for tolerance induction in VCA still remain largely unexplored.

For clinical applications, using Tregs relies on two potential approaches: (1) patient-derived *ex vivo* Treg culturing and expansion followed by reinfusion back into patients, and (2) Treg induction using IL-2/IL-2 complexes, rapamycin, and other agents that can suppress alloreactive T cells (117, 123, 124). For patient-derived Tregs, genetically modified Tregs, including antigen-specific chimeric antigen receptor (CAR)-Tregs, have been proposed for VCA. CAR-Tregs can specifically target donor cells’ MHC Class I and have previously been applied in proof-of-concept studies and clinical trials for both kidney and liver transplantation (101). Approaches to induce Treg differentiation from naïve T cells have also been recently explored in rodent VCA models. In a rat hindlimb, Fisher and colleagues designed microparticles that release TGFβ1, rapamycin, and IL-2 (TRI). This TRI-releasing microparticle treatment in combination with short-term immunosuppression resulted in allograft survival without the need for long-term immunosuppression, reduced anti-inflammatory marker expressions, and increased Treg-associated cytokines (125).

### Co-stimulatory blockade

CoB involves blocking T-cell activation and clonal expansion, and inflammatory cytokine release (126). CoB delivery through CTLA4-Ig to block T-cell co-stimulation has been developed (e.g., abatacept, belatacept) to help overcome potential immunogenic reactions (116, 127). This method alters the co-stimulation between antigen-presenting cells and T cells. For example, belatacept targets the CD28:CD80/CD86 pathway and has been clinically used in combination with low-dose immunosuppression for hand and face transplants. The mechanisms of belatacept reduce T-cell activation, alloreactivity, and help enhance allograft survival (101, 126, 127). CoB has been emerging as a promising alternative to calcineurin inhibitors previously employed in organ transplants, which have been found to have deleterious side effects. Previously, in a non-primate forearm VCA model, CoB using CTLA4-Ig showed increased allograft survival and reduced presence of donor-specific antigens relative to conventional methods (127). Combination of different tolerance induction methods where CoB, along with bone marrow transplantation and irradiation, were applied has reportedly induced long-term acceptance of VCA grafts in both rat and mouse models (128–130). CoB with bone marrow transplantation alone has also been effective in inducing mixed chimerism and tolerance in rodent models (116). In some small and large VCA models, CoB using anti-CD154 with CTLA4-Ig also increased survival (99). While effective, CoB use remains as an additional component to the existing immunosuppression regimens for VCA.

### Vascularized bone marrow transplantation

Given the multi-tissue composition of VCA grafts, a unique feature is establishing a vascularized bone marrow transplant component. Bone marrow hosts a variety of cell types including endothelial cells, fibroblasts, osteoblasts, and stromal cells. Upon transplantation, the vascularized bone marrow can produce bone marrow cells and act as a niche for HSC reconstitution. This approach can also allow a low-dose immunosuppression maintenance regimen, though the implications of such an approach have not been reported for long-term outcomes (101, 131). Using a rat hindlimb osteomyocutaneous flap model, long-term establishment of tolerance and chimerism was evident when vascularized bone marrow transplantation was coupled with tacrolimus and partial myeloablative treatment (132). Lin et al. report long-term graft survival of rat mystacial pad VCA transplantation in recipients that received vascularized bone marrow along with antilymphocyte serum, tacrolimus, and rapamycin (133). In a non-human primate partial face transplant model, a VCA graft containing vascularized bone rendered increased survival up to 430 days and without evidence of rejection, relative to a VCA graft without vascularized bone in which survival was up to only 7 days (134). The specific mechanisms underlying the potential modulatory effects that vascularized bone marrow offers have yet to be assessed; however, many of these studies show the promising role vascularized bone marrow might play in inducing tolerance in VCA grafts.

## Discussion

Currently, it is widely accepted that skin is the most immunogenic tissue in VCA, making it a prioritized target in VCA rejection studies. However, it has been demonstrated that mucosal tissue can be just as—if not more—immunogenic than skin. This contradiction provokes an interest in the many more unknowns left to explore in VCA. For instance, an additional component is considering non-skin-containing VCA grafts. While much of VCA rejection studies have focused on skin-bearing VCA grafts due to the split tolerance phenotype, extending into studies that explore immunogenicity and characterize rejection and tolerance in non-skin-bearing tissues can broaden the domain of VCA in this regard (102). The current diagnostic methods for rejection in VCA are largely dependent on the rejection of skin (e.g., visual assessment of the graft skin and the Banff classification of skin rejection). However, this will no longer offer an accurate assessment of rejection if the allograft does not contain skin. This poses the need for a diagnostic that is widely applicable to the various types of tissue within VCA. For instance, the transplantation of structures such as face, uterus, and trachea, would show signs of rejection in the mucosa, subsequently making the current diagnostic either inaccurate or inapplicable. As VCA procedures expand to include more diverse allograft structures, it may be interesting to consider non-skin-containing VCA grafts in the future. While much of VCA rejection studies have focused on skin-bearing VCA grafts due to the split tolerance phenotype, extending into studies that explore immunogenicity and characterize rejection and tolerance in non-skin-bearing tissues can broaden the scope of VCA in this regard (102). To achieve this, a more comprehensive understanding of rejection mechanisms and biomarkers is required. For example, the current description of muscle and mechanisms of vasculopathy in VCA rejection is largely based on cardiac transplantation since VCA allografts available for such studies are limited in comparison to SOT. Therefore, it would be beneficial to conduct more research into the molecular mechanisms of both acute and chronic rejection.

In terms of animal models, both small and large models have yielded considerable progress and knowledge on the immunogenicity and immune tolerance mechanisms in VCA as outlined in the previous sections. A well-recognized challenge is the significant difference between small and large VCA models where translation from small animals to pre-clinical-relevant models or clinical scale has been unsuccessful. Protocols for applying calcineurin inhibitors and donor stem cell transplantation, for example, showed promising outcomes in mice but failed in applying tolerance induction for larger models (135). Some considerations for future studies include recognizing that allo-specific memory B and T cells can form in both large animal and humans due to prior antigen exposure, whereas this feature is not observed in mice. High amounts of alloreactive memory T cells are found in primates as opposed to mice (136). Expression of MHC Class II antigens are also varied between large animals and humans relative to rodents, indicating that many protocols must be validated in their translation from rodent studies to larger models prior to considering for clinical applications (135). Despite the disadvantages, small models offer technical feasibility and establishing potential proof-of-concept evidence. Large animal models hold pre-clinical relevance and have closer alignment with humans in terms of anatomy.

Tolerance induction for VCA has been a rapidly developing area where multiple methods have been tested. The use of both cell-based and pharmaceutical-based approaches shows promise. Many methods—such as co-stimulatory blockade, T-cell depletion, Treg modulation, vascularized bone marrow transplantation, and delayed tolerance—have been used in various combinations to maximize allograft survival. The multi-component approach to induce tolerance reflects the complexity of tolerance induction for multi-tissue VCA grafts, different from solid organs. While much progress has been made for tolerance induction methods for VCA, there are many areas of further investigation required before its clinical applications. The use of exosomes presents an exciting future perspective for tolerance induction. These extracellular vesicles can potentially act as markers of graft failure for both solid organ transplants and VCAs given that they release genetic, protein, and lipid content post-transplantation. Additional studies have also examined exosomes in both donor and recipients to identify their role in moderating tolerance and graft rejection (137). Studies using rodent models have shown that exosomes derived from donor stem cells increase VCA graft survival times, show no rejection signs, and increase donor cell chimerism (138, 139). Mesenchymal stem cells and adipose-derived stem cells have been of considerable interest for their immunomodulatory properties (140).

Many protocols exclusively use radiation as part of their conditioning regimens. Myeloablation methods can pose risks for patients and give rise to potential hematological and infective complications when applied clinically (81). Given that VCA grafts are derived from deceased donors, tolerance induction methods will also require further work in reducing pre-conditioning times than would be used for solid organ transplantations that can otherwise rely on a living donor pool. Successful tolerance induction for VCA may entail using immunosuppression short-term, low toxicity for patients, and same-day or minimal time for pre-conditioning (126). In the ultimate goal of achieving immune tolerance, while much work remains in both basic science and clinical protocol development, the future of VCA looks promising.
